# Beyond Low Back Pain! The Influence of Physical Activity on Mental Health, Reflected in the Functionality of People with Low Back Pain

**DOI:** 10.3390/healthcare13121471

**Published:** 2025-06-18

**Authors:** Franciele Parolini, Klaus Becker, Márcio Goethel, Ricardo J. Fernandes, Hélder Fonseca, Ulysses F. Ervilha, João Paulo Vilas-Boas, Rubim Santos

**Affiliations:** 1 Center for Rehabilitation Research (CIR), ESS, Polytechnic of Porto, Rua Dr. António Bernardino de Almeida, 4249-015 Porto, Portugal; rss@ess.ipp.pt; 2 Porto Biomechanics Laboratory, Center of Research, 4200-450 Porto, Portugal; klausmagnobecker@gmail.com (K.B.); gbiomech@fade.up.pt (M.G.); ricfer@fade.up.pt (R.J.F.); ulyervil@usp.br (U.F.E.); jpvb@fade.up.pt (J.P.V.-B.); 3 Center of Research, Education, Innovation and Intervention in Sport, Faculty of Sport, University of Porto, 4200-450 Porto, Portugal; 4 Research Centre in Physical Activity, Health, and Leisure (CIAFEL), Faculty of Sport, University of Porto, Rua Dr. Plácido Costa, 4200-450 Porto, Portugal; hfonseca@fade.up.pt; 5 Laboratory of Physical Activity Sciences, School of Arts, Sciences and Humanities, University of São Paulo, São Paulo 05508-220, SP, Brazil

**Keywords:** physical activity, mental health, low back pain, canonical correlation

## Abstract

Background/Objectives: Psychological factors play a crucial role in the experience of acute low back pain and may influence functional outcomes. However, the interplay between these factors and levels of physical activity remains poorly understood. Methods: This cross-sectional observational study examined the relationship between psychological variables and functional disability in individuals with acute low back pain, considering different levels of physical activity. Data were collected from 1021 participants through an online platform between 8 June 2022 and 8 April 2023. Standardized instruments were used to assess functional limitations, emotional distress (depression, anxiety, and stress), daily pain catastrophizing, and physical activity levels. A canonical correlation analysis was conducted to explore the multivariate associations between psychological and functional variables. Results: There was a statistically significant association between higher levels of emotional distress and greater functional impairment related to low back pain. This association was observed in both the light-physical-activity group (canonical coefficient = 0.266; *p* = 0.017), the moderate-physical-activity group (0.237; *p* = 0.092), and the vigorous-physical-activity group (0.177; *p* = 0.013). Participants engaging in vigorous physical activity exhibited more favorable psychological profiles and lower functional disability compared to those with lower levels of activity. Conclusions: Regular and vigorous physical activity appears to be a protective factor for mental health and may help reduce functional disability in individuals with acute low back pain. These findings underscore the importance of considering physical activity levels when addressing psychological and functional outcomes in this population.

## 1. Introduction

Low back pain is recognized in many countries as the leading cause of musculoskeletal complaints, with significant health and economic impacts due to the limitations and disabilities that it imposes on individuals [1]. This condition leads to increased healthcare use and high rates of absenteeism due to pain, adversely affecting patients’ quality of life, burdening healthcare systems, and reducing their productivity [1,2]. The pain process encompasses sensory, cognitive, and affective components, the latter including feelings of annoyance, sadness, anxiety, and depression in response to a harmful stimulus [3,4,5]. This is corroborated by the revised definition of the International Association for the Study of Pain (IASP), which conceptualizes pain as an unpleasant sensory and emotional experience associated with, or similar to, actual or potential tissue injury [1].

The IASP definition was recently tested through a mathematical model utilizing functionality, symptoms of depression, anxiety, stress, and pain catastrophizing data via artificial neural network to identify patterns [6]. The results confirmed the direct association between psychological factors and pain, indicating that mental health aspects can exacerbate pain and impair individual functionality. Accordingly, pain must be understood within a biopsychosocial framework that integrates biological, psychological, and social dimensions [7,8].

Psychological factors have become increasingly prevalent among individuals with low back pain, with an individual’s mental health status fluctuating alongside daily variations in symptom intensity and other contextual factors [9,10]. Concurrently, the role of physical activity as a multifaceted intervention influencing biological, psychological, and social mechanisms has received increasing attention [11,12]. Recent evidence indicates that physical activity significantly reduces symptoms of anxiety, depression, and stress [13,14], thereby reinforcing its role in improving mental health outcomes. While regular physical activity is linked to a reduced risk of depression, its relationship with anxiety symptoms is less clear, warranting further investigation into the optimal intensities and durations to positively influence mental health [9,15,16].

Despite numerous studies addressing the interaction between exercise and psychological factors in chronic low back pain and their effects on functionality [17,18,19,20], significant gaps remain regarding the acute phase of low back pain. Acute low back pain differs substantially from the chronic condition in its clinical presentation and psychosocial dynamics, which may affect recovery trajectories and treatment responses [21,22,23,24]. Furthermore, studies incorporating multivariate analytical approaches that simultaneously consider psychological factors, functionality, and physical activity intensity in acute low back pain populations are scarce.

Therefore, this study aims to analyze the relationships between psychological factors and individual functionality in individuals experiencing acute low back pain, considering different intensities of physical activity. Understanding these complex interactions will contribute to addressing a critical knowledge gap and support the development of tailored interventions. We hypothesize that higher levels of emotional distress, including symptoms of depression, anxiety, and stress, are associated with greater functional impairment in individuals with acute low back pain. Furthermore, we propose that the intensity of physical activity moderates this relationship, such that engagement in moderate to high levels of physical activity attenuates the negative impact of psychological distress on functional outcomes.

## 2. Materials and Methods

This was a cross-sectional observational study, which was approved by the ethics committee of the School of Health of the Polytechnique of Porto (CE0092B), and the procedures were conducted in accordance with the guidelines of the Declaration of Helsinki. A total of 1.208 volunteers consented to participate in the study through an online informed consent form. Participants were recruited via online advertisements, disseminated through social media platforms, academic forums, and institutional mailing lists. Of these, 1.021 participants were included in the final analysis. A total of 187 individuals were excluded for not meeting the inclusion criteria (e.g., age outside 18–35 years, absence of low back pain as the main complaint, not engaging in physical activity, incomplete responses, or a failure to submit the survey).

The presence of acute low back pain was self-reported and defined as at least one episode of pain in the lumbar region within the previous six weeks, consistent with established definitions of acute low back pain. No clinical evaluation was performed; participants completed the survey independently and voluntarily via an online platform. The inclusion criteria were being between 18 and 35 years old, reporting low back pain as the main complaint, engaging in physical activity, completing all four questionnaires, and understanding the instructions for completing the survey.

### 2.1. Data Collection

The survey examining the relationship between low back pain and psychological variables was developed using an online platform. It comprised four questionnaires: (i) the Oswestry disability index to assess the functional impact of low back pain; (ii) the depression, anxiety, and stress scale to evaluate psychological symptoms; (iii) the daily pain catastrophizing questionnaire to measure the intensity of pain-related thoughts; and (iv) the international physical activity short-form questionnaire to assess physical activity levels. Data were collected through self-administered online responses between 8 June 2022 and 8 April 2023. Participants also provided demographic and clinical information, including gender, body mass, age, height, sociodemographic status, psychiatric diagnoses, and the frequency of low back pain episodes in the previous 42 days.

The final sample included 1.021 participants (63% women, 37% men) who met the inclusion criteria. Among them, 20% reported diagnosed spinal conditions: scoliosis (15%), disk protrusion (3%), herniated disk (1%), and anterolisthesis (1%). All participants had experienced at least one acute episode of low back pain in the previous six weeks. The distribution of episodes was as follows: 10% had 1 episode (102 participants), 20% had 10 (204), 30% had 14 (306), 15% had 20 (153), 10% had 25 (102), 5% had 30 (51), and 10% had more than 30 episodes (102). Recruitment aimed for broad inclusion to capture a comprehensive picture of the relationship between low back pain and psychosocial and functional variables.

Participants were divided into three groups based on their physical activity intensity. Group 1 (light activity) comprised 256 participants, who had a mean age of 23.5 ± 6.2 years, body mass of 64.8 ± 13.5 kg, and height of 166.8 ± 8.4 cm. Group 2 (moderate activity) comprised 236 participants, who had a mean age of 23.5 ± 5.0 years, body mass of 65.4 ± 14.4 kg, and height of 166.3 ± 12.8 cm. Group 3 (vigorous activity) comprised 529 participants, who had a mean age of 23.5 ± 5.9 years, body mass of 65.2 ± 15.4 kg, and height of 167.5 ± 12.8 cm. The average number of days with low back pain in the 42-day period was 7.9 ± 9.6 in group 1, 6.9 ± 9.1 in group 2, and 6.9 ± 8.7 in group 3.

### 2.2. Instruments

The Oswestry disability index was used to measure the impact of back pain on daily living activities, particularly regarding pain intensity, lifting weights, social interaction, sitting, standing, traveling, sex life, sleeping, walking, and personal care [2,25]. It is composed of ten questions with six alternatives (each ranging in scores from 0 to 5). The first question assesses the intensity of pain, while the others score the pain’s impact on daily activities (such as personal care, lifting weights, walking, sitting, standing, sleeping, social activities, and mobility). The total is obtained by multiplying the sum of the scores by five and then dividing the result by the total number of questions answered. The score is classified a minimal, moderate, and severe disabilities (0–20, 21–40, and 41–60%, respectively), disabled (61–80%), and bedridden (81–100%).

The short-form depression, anxiety, and stress scale [26] included 21 items and was designed to assess depression, anxiety, and stress domains (each one being represented by seven items). Participants rated each item on a 0–3 scale from did not apply to me at all to applied to me very much or most of the time. Each domain is represented by a subscale score (the sum of the item responses for that subscale multiplied by two to be comparable with the original 42-item depression, anxiety, and stress scale). This instrument was previously validated and considered reliable [26], with a high score representing worse depression, anxiety, or stress. Cut-off points for normal, mild, moderate, severe, and extremely severe classifications, based on population norms, are provided. Classification symptoms are rated as 0–10 (normal), 11–18 (mild), 19–26 (moderate), 27–34 (severe), and 35–42 (extremely severe) for stress; 0–6 (normal), 7–9 (mild), 10–14 (moderate), 15–19 (severe), and 20–42 (extremely severe) for anxiety; and 0–9 (normal), 10–12 (mild), 13–20 (moderate), 21–17 (severe), and 28–42 (extremely severe) for depression.

The daily pain catastrophizing scale (d-PCS) [8] is a 14-item questionnaire, designed to assess pain catastrophizing over the past 24 h, with responses rated on a Likert scale from 0 (“never”) to 4 (“always”). The total score is calculated as the sum of all items (range of 0–56), with higher scores indicating greater catastrophizing. For use in this study, the instrument was translated and culturally adapted into Portuguese following rigorous methodological guidelines, provided by the International Test Commission [27]. The use of the d-PCS enables enhanced analytical precision in research on adaptive pain mechanisms.

The IPAQ-SF consists of seven items and is straightforward to administer in clinical settings. It collects data on the frequency (days per week) and duration (minutes per day) of walking and moderate and vigorous physical activities, as well as sedentary time, over the previous seven days. Based on these responses, a categorical score classifies individuals’ physical activity levels as low, moderate, or vigorous, according to their total weekly energy expenditure, expressed in MET-minutes, which integrates both the intensity and duration. In the present study, participants were grouped into three physical activity levels based on internationally recognized IPAQ scoring protocol thresholds: light (600–1499 MET-min/week), moderate (1500–2999 MET-min/week), and vigorous (≥3000 MET-min/week) [28,29]. These cut-off points are widely accepted and validated in population-based research to standardize physical activity classification, enabling comparability across studies. The IPAQ-SF is extensively validated for its reliability and construct validity in estimating physical activity in diverse populations, making it a robust tool for epidemiological and clinical research. Using MET-minutes as the basis for classification offers a standardized and internationally endorsed metric to evaluate physical activity’s impact on health outcomes, including cardiovascular and metabolic parameters [29].

### 2.3. Statistical Analysis

All statistical analyses were conducted using SPSS (version 27), adopting a two-tailed significance level of *p* ≤ 0.05. Descriptive statistics were first computed to characterize the sample, including the means, standard deviations, and frequency distributions of sociodemographic and clinical variables. To examine the multivariate associations between psychological symptoms and low back pain outcomes, a canonical correlation analysis (CCA) was performed. Two sets of variables were included in the analysis:

- Set 1: Mental Illness—comprising stress, anxiety, and depression, as assessed by the respective subscales of the depression, anxiety, and stress scale.
- Set 2: Low Back Pain—comprising pain intensity (numeric pain rating scale), functional disability, Oswestry disability index, number of low back pain episodes in the past six weeks, and daily pain catastrophizing.

The CCA allowed for the identification of canonical functions that maximized the correlation between linear combinations of the two sets. Canonical coefficients, structure coefficients, and explained variance were computed and interpreted. This method was chosen due to its robustness in detecting multivariate relationships, particularly in complex clinical conditions such as chronic low back pain [30,31,32,33]. In addition, a multivariate analysis of variance (MANOVA) was conducted to compare psychological symptom levels (stress, anxiety, and depression) across the groups, defined by physical activity intensity (light, moderate, and vigorous). MANOVA was selected to account for the potential intercorrelations among dependent variables while minimizing type I errors.

Mediation analyses were also performed using the PROCESS macro for SPSS (model 4), with 5000 bootstrap samples and bias-corrected confidence intervals. Physical activity intensity was entered as the independent variable, psychological symptoms (stress, anxiety, and depression) as mediators, and functional disability as the dependent variable. The significance of indirect effects was used to determine the presence of statistical mediation. Finally, effect sizes and statistical power were estimated using G*Power 3.1.7, following [34] conventions: small (>0.20), medium (>0.50), and large (>0.80) effects.

## 3. Results

The mediation analysis indicated that the total number of days of physical activity was not significantly associated with psychological health (β = −0.2547; *p* = 0.234) or with functional disability, as assessed by the ODI (β = 0.0621; *p* = 0.459). The confidence intervals of these coefficients included zero, reinforcing the absence of robust evidence of association. On the other hand, psychological symptoms, represented by stress, anxiety, and depression, showed a positive association with functional disability (β = 0.0466; *p* < 0.001), revealing that higher levels of psychological distress are statistically associated with greater functional limitation in individuals with acute low back pain.

**Figure 1:**
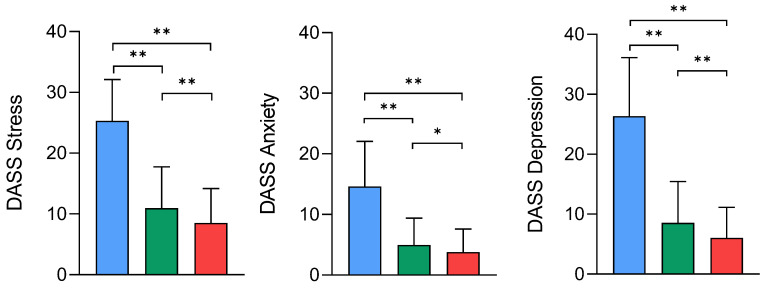
Canonical correlations between psychological and functional variables across low- (blue), moderate- (green), and vigorous-physical-activity (red) groups. * *p* < 0.05; ** *p* < 0.01.

Figure 1 depicts the statistically significant differences in stress, anxiety, and depression levels across the three physical activity intensity groups. Participants engaging in vigorous physical activity consistently exhibited the lowest levels of these psychological symptoms, while those in the light-activity group presented the highest levels. The moderate-activity group demonstrated intermediate symptom severity; however, statistical comparisons did not yield consistent significant differences for this group (*p* > 0.05), suggesting a more variable psychological profile relative to the other intensity levels.

The mean stress levels were highest in the light-physical-activity group (mean 25.3 ± 6.8), followed by the moderate-activity-group (10.9 ± 6.7), and lowest in the vigorous-activity group (8.5 ± 5.6). A similar pattern was observed for anxiety, with mean values of 14.6 ± 7.4 in the light-activity group, 4.9 ± 4.4 in the moderate-activity group, and 3.7 ± 3.8 in the vigorous-activity group. Depression levels followed the same trend, with higher mean scores in the light-activity group (26.3 ± 9.7) compared to the moderate- (8.5 ± 6.9) and vigorous-activity groups (6.0 ± 5.1). These findings indicate an inverse association between the physical activity intensity and psychological distress levels. To quantify the magnitude of differences in psychological symptom levels across physical activity intensity groups, Cohen’s d effect sizes were calculated for stress, anxiety, and depression. Very large effect sizes were observed when comparing the light-activity group to both the moderate- and vigorous-activity groups, particularly for depression (d = 2.10 and d = 2.93, respectively) and stress (d = 2.13 and d = 2.79). Anxiety levels also showed large differences between the light-activity group and both the moderate- (d = 1.58) and vigorous (d = 2.08)-activity groups. These results highlight meaningful psychological differences across physical activity intensity levels, beyond statistical significance.

### Canonical Correlation of Psychological Symptoms and Functionality of Individuals with Low Back Pain in Different Physical Activity Intensity Groups

Two pairs of canonical variables were extracted for each physical activity intensity group (light and vigorous). The first pair in both groups demonstrated statistically significant canonical correlations, indicating a meaningful multivariate relationship between the sets of variables analyzed. In contrast, the second pair did not reach statistical significance in either group. For the moderate-activity group, neither pair of canonical variables exhibited significant correlations. These findings suggest that physical activity intensity is associated with distinct patterns of relationships among the variables, as reflected by the variation in canonical correlation coefficients and significance levels across groups (see Table 1).

Significant pairs of canonical variables (*p* < 0.05) were observed in the light- and vigorous-physical-activity groups, which were analyzed in depth. The moderate-activity group did not show statistical significance. The two pairs of canonical variables extracted were labeled based on their factor loadings: psychological distress (depression, anxiety, and stress) and low back pain (functional disability, pain episodes, and catastrophizing). These labels are descriptive and based on the composition of the canonical loadings and are not causal inferences.

**Figure 2:**
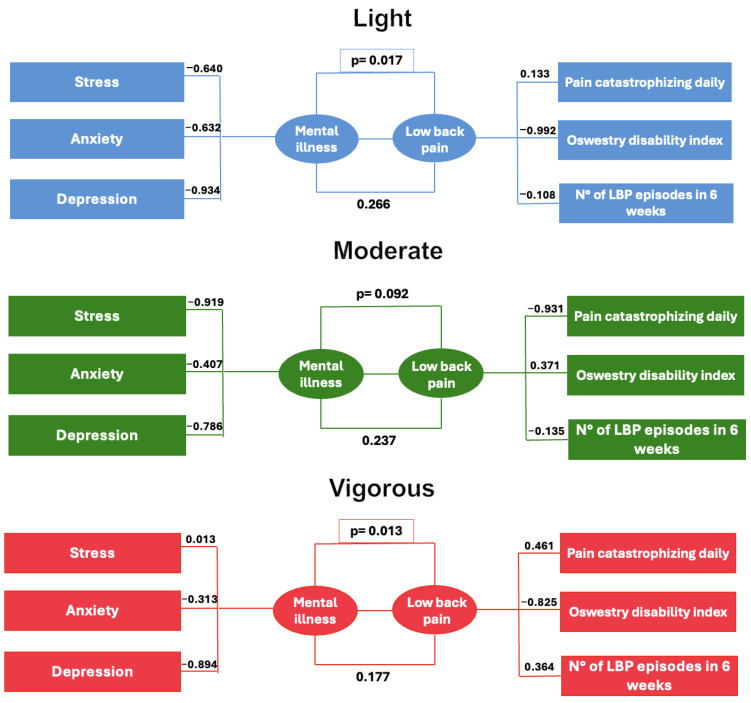
Canonical loadings of psychological and functional variables by physical activity intensity. Loadings reflect the contribution of each variable to the identified canonical functions.

In Figure 2, within the light-physical-activity group, depression exhibited the highest factor loading (−0.934), followed by anxiety (−0.632) and stress (−0.640), indicating a strong negative contribution of psychological distress to the respective dimensions. Catastrophizing demonstrated the greatest influence on the low back pain axis with a loading of −0.992, whereas functional disability showed a modest positive loading (0.133), and the number of episodes contributed weakly (−0.108). In the vigorous-activity group, depression also displayed a substantial loading (−0.894), albeit slightly lower than in the light-activity group. Anxiety presented a lower loading (−0.313), and stress showed minimal contribution (0.013). Notably, catastrophizing exhibited a positive loading (0.461), reflecting a distinct pattern compared to the light-activity group. Functional disability was associated with a significant negative loading, while the number of episodes had a positive loading (0.364). These factor loadings illustrate differential patterns of association among psychological and clinical variables across physical activity intensity levels.

Table 2 shows the explanatory power of key variables across physical activity intensity levels. The variance explained by psychological distress is higher in the light- (56.1%) and moderate (54.1%)-activity groups compared to the vigorous-activity group (29.9%). In contrast, the variance explained by low back pain remains relatively constant across all groups, ranging from 33.8% to 34.3%. These results suggest that psychological distress accounts for a larger proportion of variance in groups with lower physical activity intensity, while the influence of low back pain is stable, regardless of activity level.

## 4. Discussion

This study revealed that elevated levels of pain catastrophizing and depression are associated with poorer functional performance in individuals with acute low back pain prevalent and disabling conditions. Canonical correlation analysis (CCA), a multivariate statistical method, was employed to simultaneously explore multiple psychosocial and functional variables, enabling the identification of complex patterns of association that would likely go undetected using traditional univariate approaches. The CCA highlighted significant interrelationships among physical activity intensity, psychological symptoms, and functional status, demonstrating that clusters of factors such as depression and stress jointly influence levels of functional disability. In contrast to conventional regression models, which assess isolated relationships, CCA provides an integrated perspective on psychosocial influences, capturing the dynamic and multifactorial nature of low back pain [30,31,35].

Participants in the study reported at least one episode of low back pain within the previous six weeks, and the findings offer a deeper understanding of how psychological factors and physical activity intensity interact to modulate the clinical experience of acute pain. When analyzing subgroups based on the type of physical activity, it was observed that among participants engaging primarily in light-intensity physical activity, depression was significantly associated with functional status. This suggests that individuals with more severe depressive symptoms perceive greater functional impairment.

This finding reinforces the role of depression not only as a factor related to increased pain perception, but also as a contributor to functional limitations, an aspect that is important for the effective management of acute low back pain [23,36]. Although the literature on chronic pain has already established this relationship [4,11,23,24], the present findings extend this knowledge to acute episodes, highlighting the role of psychological factors in both functional outcomes and the suitability of physical activity intensity. Anxiety and stress, although exhibiting smaller effect sizes, were also found to be associated with functional status, suggesting that psychological symptoms may directly influence the degree of functional impairment, rather than merely reflecting pain perception [37,38]. This observation underscores the need for multidisciplinary approaches that address these factors to enable more effective interventions [39].

A key contribution of this study is the differentiation of groups based on their physical activity intensity. In the vigorous-activity group, depression remained associated with functional status, although with a moderate effect size, suggesting that the relationship between psychological symptoms and functionality may vary according to the level of physical activity [20,40,41]. Moreover, the analysis of daily pain catastrophizing in the vigorous-activity group revealed a positive association with perceived disability. This finding suggests that among individuals who engage in high-intensity physical activity, catastrophizing may contribute to greater perceived functional limitations related to pain [20,42]. A possible explanation for this phenomenon lies in the effects of physical overload or overtraining, which may be interpreted more negatively and perceived as more limiting by individuals with high levels of catastrophizing [43].

This apparent contradiction, where vigorous physical activity may be associated with psychological benefits but also with increased levels of catastrophizing, warrants attention, as it suggests that the effect of physical activity on the pain experience may be modulated by specific psychological factors, such as the tendency to catastrophize [4,10,44,45]. It is important to emphasize that this process does not imply that pain or physical exertion are imaginary; rather, it refers to how individuals interpret and respond to nociceptive signals. A negative interpretation of these signals may lead to the avoidance of physical activity and the underestimation of one’s own functional capacity, thereby influencing perceived limitations [43,45].

The beneficial effects of vigorous physical activity on psychological symptoms may be partially explained by neurophysiological and behavioral mechanisms. Evidence suggests that engaging in high-intensity exercise is associated with the release of endorphins, which help reduce pain perception and enhance mood [46]. In addition, physical activity can function as a cognitive distraction by redirecting attention away from pain and increasing activation in brain regions that are involved in pain modulation, particularly during tasks that require cognitive distraction [47]. It can also promote positive social interactions, thereby enhancing psychological well-being [48]. The integration of these factors is essential for understanding individual variations in pain response and the associated functional impact.

The mediation analysis indicated that the total number of days of physical activity was not significantly associated with psychological health or lumbar spine functionality, as measured by the ODI [2]. In line with the literature, which supports our findings, we suggest that this relationship is more complex and influenced by factors such as activity intensity, psychological variables, and overall physical function, rather than by the total number of days of physical activity alone. The significant association between psychological symptoms (stress, anxiety, and depression) and lumbar functional disability underscores the importance of psychosocial factors in the experience of acute low back pain. This reinforces the notion that, in the context of acute low back pain, interventions should go beyond the simple prescription of exercise and incorporate psychological strategies as an integral part of therapy [24,43].

Therefore, the early identification of these factors can guide more appropriate psychosocial interventions, such as cognitive behavioral therapy, pain education, and individualized exercise prescription [32,39]. This observation suggests that rehabilitation programs for acute low back pain may benefit from interventions that consider not only exercise intensity but also individual psychological characteristics, particularly pain catastrophizing, in order to maximize therapeutic outcomes [49].

### 4.1. Clinical Implications and Future Directions

Although this study does not allow for causal inferences, it provides important insights into how psychological factors and the volume and intensity of physical activity interact in patients with acute low back pain. The combination of psychological interventions with personalized physical activity appears to be a promising strategy for managing acute low back pain. The attenuated impact of vigorous physical activity on psychological symptoms suggests that interventions may be beneficial for some individuals [20,40,41], but attention must be given to catastrophizing behavior to avoid pain amplification [4,44]. Furthermore, the absence of a significant association between the volume of physical activity, psychological health, and lumbar spine functionality suggests that future studies should explore how different types of exercise and intensities (aerobic, resistance, flexibility) interact with psychological factors in modulating pain and functional capacity in acute low back pain, as the literature still presents gaps in this area [21,22,50].

### 4.2. Limitations

This study has some limitations. Its cross-sectional and observational design prevents the establishment of causal relationships between the analyzed variables. Additionally, the use of self-reported data through questionnaires may introduce response and perception biases. The sample, consisting of individuals with acute low back pain, may not fully capture the complexities of chronic pain, in which the interactions between psychological symptoms and pain tend to be more dynamic and multifaceted. Moreover, the study population was limited to young adults aged 18–35 years, which restricts the generalizability of the findings to older age groups. This age group was intentionally selected, because it is less frequently affected by chronic low back pain, allowing for a more targeted analysis of acute pain episodes. These episodes often present different biopsychosocial mechanisms than those found in chronic conditions [23,24]. Future longitudinal studies and randomized clinical trials will be essential to more robustly examine the impact of psychosocial factors and physical activity intensity, as well as to explore how these elements can be effectively integrated into the management of acute low back pain, not only to promote recovery but also to reduce the recurrence of future pain episodes.

## 5. Conclusions

The current study reinforces the complex interaction between physical activity, psychological factors, and functionality in acute low back pain, as demonstrated by canonical correlation analysis. The findings indicate that individuals engaging in vigorous physical activity tend to report better psychological profiles and less functional disability. This suggests that not just the quantity, but especially the intensity of physical activity plays a crucial role in rehabilitation outcomes. Personalized therapeutic strategies that integrate physical and psychological assessments are essential to optimizing care for individuals with acute low back pain.

## Figures and Tables

**Table 1:** Physical activity groups with pairs of canonical correlations and significance test.

Group Physical Activity	Canonical Correlation Pair	Canonical Correlation Coefficient	Significance Test		
			Wilks	f Value	*p*-Value
Light	1	0.266	0.923	2.254	0.017
	2	0.078	0.994	0.389	0.816
Moderate	1	0.237	0.937	1.674	0.092
	2	0.079	0.993	0.403	0.807
Vigorous	1	0.177	0.961	2.340	0.013
	2	0.084	0.992	1.084	0.363

Legend: The numbers 1 and 2 refer to the first and second canonical variate pairs extracted from the canonical correlation analysis, ordered by the strength of the canonical correlation.

**Table 2:** Explanatory power of typical variables, expressed in percents (%).

Level of Physical Activity	Mental Illness%	Low Back Pain%
Light	56.1	33.8
Moderate	54.1	34.2
Vigorous	29.9	34.3

## Data Availability

The data presented in this study are available on request from the corresponding author. The data are not publicly available due to privacy.
